# Machine-learning guided discovery of a new thermoelectric material

**DOI:** 10.1038/s41598-019-39278-z

**Published:** 2019-02-26

**Authors:** Yuma Iwasaki, Ichiro Takeuchi, Valentin Stanev, Aaron Gilad Kusne, Masahiko Ishida, Akihiro Kirihara, Kazuki Ihara, Ryohto Sawada, Koichi Terashima, Hiroko Someya, Ken-ichi Uchida, Eiji Saitoh, Shinichi Yorozu

**Affiliations:** 1 Central Research Laboratories, NEC Corporation, Tsukuba, 305-8501 Japan; 2 PRESTO, JST, Saitama, 322-0012 Japan; 3 Department of Materials Science and Engineering, University of Maryland, College Park, MD 20742 USA; 4 Center for Nanophysics and Advanced Materials, University of Maryland, College Park, MD 20742 USA; 5 National Institute of Standards and Technology, Gaithersburg, MD 20899 USA; 6 Research Center for Magnetic and Spintronic Materials (CMSM), National Institute for Materials Science (NIMS), Tsukuba, 305-0047 Japan; 7 Research and Services Division of Materials Data and Integrated System (MaDIS), National Institute for Materials Science (NIMS), Tsukuba, 305-0047 Japan; 8 Institute for Materials Research, Tohoku University, Sendai, 908-8577 Japan; 9 Center for Spintronics Research Network, Tohoku University, Sendai, 980-8577 Japan; 10 Advanced Institute for Materials Research, Tohoku University, Sendai, 908-8577 Japan; 11 Advanced Science Research Center, Japan Atomic Energy Agency, Tokai, 319-1195 Japan

## Abstract

Thermoelectric technologies are becoming indispensable in the quest for a sustainable future. Recently, an emerging phenomenon, the spin-driven thermoelectric effect (STE), has garnered much attention as a promising path towards low cost and versatile thermoelectric technology with easily scalable manufacturing. However, progress in development of STE devices is hindered by the lack of understanding of the fundamental physics and materials properties responsible for the effect. In such nascent scientific field, data-driven approaches relying on statistics and machine learning, instead of more traditional modeling methods, can exhibit their full potential. Here, we use machine learning modeling to establish the key physical parameters controlling STE. Guided by the models, we have carried out actual material synthesis which led to the identification of a novel STE material with a thermopower an order of magnitude larger than that of the current generation of STE devices.

## Introduction

Waste heat is ubiquitous in modern society, and thermoelectric technologies based on the Seebeck effect have been embraced as a central avenue to a sustainable future^1–3^. Unfortunately, conventional thermoelectric (TE) devices suffer from high fabrication cost due to their complex structure, in which p-type and n-type thermoelectric materials are cascade-connected in an alternating way. The emergence of novel thermoelectric devices based on the spin-driven thermoelectric (STE) phenomena offers a potential solution to this problem. In contrast to the conventional TE devices, the STE devices consist of simple layered structures, and can be manufactured with straightforward processes, such as sputtering, coating and plating, resulting in lower fabrication costs^4^. An added advantage of the STE devices is that they can double their function as heat-flow sensors, owing to their flexible structures and lower thermal resistance^5^.

STE devices utilize the spin-Seebeck effect (SSE)^6–10^ and the anomalous Nernst effect (ANE)^11–13^. SSE generates a spin current from a temperature gradient in a magnetic material. By connecting a metallic film having large spin-orbit interaction (such as Pt) to a magnetic material, one can convert the spin current into the electrical current via the inverse spin-Hall effect (ISHE)^14–17^. Thermoelectric conversion based on SSE can lead to an entirely new class of inexpensive and versatile thermoelectric devices^4^. Unfortunately, advance in device development is hampered by the lack of understanding of the fundamental mechanism behind SSE and the materials parameters governing it. Several different theories have been put forth to explain the phenomenon^18,19^, but a unified picture of its mechanism is yet to emerge. Key materials parameters driving SSE have not been identified to date, and there are no clear pathways to enhance the thermopower and related figures of merit.

But in fields in which basic understanding is still too unreliable to guide progress, data-driven approaches using statistics modeling and machine learning can be employed to uncover hidden links and correlations. Machine learning methods are becoming indispensable tools for study of materials (for recent review see, for example, refs^20,21^). These methods are now routinely used to address many key materials science questions. In particular, machine learning informed models are utilized to search for new materials, including potential magnets^22^, ferroelectrics^23^ and superconductors^24^. Although material synthesis guided by machine learning has been relatively rare so far^25^, it is very likely going to become a commonplace in the future.

Utilizing these methods, we have developed a systematic approach to uncovering the major materials variables governing the SSE. We combine machine learning modeling with high-throughput experimentation, and use modeling results for designing combinatorial libraries^26–30^. We have successfully leveraged the machine-learning-informed knowledge of the dependence of SSE on materials parameters to arrive at a novel and high-performance STE material utilizing ANE, which converts a heat current into an electrical current via the spin-orbit interaction in a single ferromagnetic material. Out of a number of proposed materials systems, a composition spread of one ternary system has led to the identification of Fe_0.665_Pt_0.27_Sm_0.065_, which exhibits thermopower as large as 11.12 μV/K.

## Results

### Material data collection

**Figure 1:**
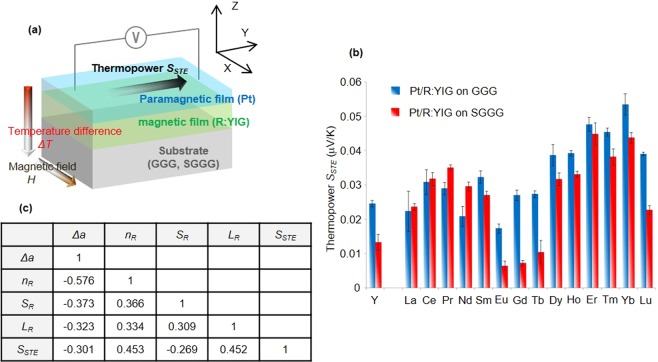
Data of spin-driven thermoelectric materials. **(a)** Schematic of the spin-driven thermoelectric (STE) device using spin-Seebeck effect (SSE) consisting of a Pt layer, a rare-earth substituted yttrium iron garnet (R_1_Y_2_Fe_5_O_12_, referred to as R:YIG) layer and a (111)-oriented Gadolinium Gallium Garnet (Gd_3_Ga_5_O_12_, referred to as GGG) substrate or a (111)-oriented Substituted Gadolinium Gallium Garnet (Gd_2.675_Ca_0.325_Ga_4.025_Mg_0.325_Zr_0.65_O_12_, referred to as SGGG) substrate. When a temperature difference Δ*T* and a magnetic field *H* are applied along the z and x direction, respectively, one can detect the thermopower *S*_*STE*_ along the y direction. (**b**) Data of the thermopower *S*_*STE*_ for different rare-earth substituted YIG (R:YIG). The magnitude of *S*_*STE*_ varies depending on the choice of rare-earth element. Error bars are standard deviation. (**c**) Pearson correlation coefficient (PCC) matrix. The values with respect to *S*_*STE*_ are less than 0.5.

First, we performed experiments to collect STE data for various materials. Figure 1a shows the general configuration of one of the STE devices using the spin-Seebeck effect (SSE). It is composed of a paramagnetic conductive layer, a magnetic layer, and a single crystal substrate. We adopted a bilayer consisting of platinum (Pt) and rare-earth-substituted yttrium iron garnet (R_1_Y_2_Fe_5_O_12_, referred to as R:YIG), where R stands for a rare-earth element. When a temperature difference Δ*T* and a magnetic field *H* are applied along the z and the x direction, respectively, one can detect the thermopower *S*_*STE*_ along the y direction. Therefore, this device converts electric energy into thermal energy. The details of the STE device are shown in the Supplementary Information 1.

The substitution in R:YIG with different rare-earth elements changes the physical properties of the material, and allows us to study the impact this has on the STE phenomena. Figure 1b shows the thermopower measured for Pt/R:YIG samples, fabricated on Gd_3_Ga_5_O_12_ (GGG) and Gd_2.675_Ca_0.325_Ga_4.025_Mg_0.325_Zr_0.65_O_12_ (SGGG) substrates and with various rare-earth elements R - La, Ce, Pr, Nd, Sm, Eu, Gd, Tb, Dy, Ho, Er, Tm, Yb, and Lu (Pm, which is radioactive, was excluded from the study). It is clear that *S*_*STE*_ strongly depends on the choice of the R element. Differences in measured *S*_*STE*_ can be dramatic; for example, the response of Pt/Yb:YIG on GGG is about three times as large as that of Pt/YIG on GGG.

The striking dependence of *S*_*STE*_ on the choice of R suggests that the physical parameters of R strongly influence STE. To expose and quantify this connection we employed machine learning. As a first step, we considered different descriptors that can encode the properties of different rare-earth elements, such as atomic weight *n*_*R*_, spin and orbital angular momenta *S*_*R*_ and *L*_*R*_, lattice mismatch Δ*a* between R:YIG and the substrate, number of unfilled orbitals, elemental melting temperatures, magnetic moments, and ground state volumes, etc. It is, however, difficult to experimentally isolate and extract the *S*_*STE*_ dependence on a given physical parameter of R. To delineate the relation between the atomic weight *n*_*R*_ and *S*_*STE*_, for instance, it would be necessary to measure the *S*_*STE*_ for different *n*_*R*_ while keeping all other predictors fixed, which is experimentally not feasible. In order to uncover the subtle correlations and the physical origin of the STE hidden in the initial experimental results, we first calculated Pearson correlation coefficient (PCC) [shown in Fig. 1(c)]. There appears to be roughly linear relationship between Δ*a*, *n*_*R*_, *S*_*R*_, *L*_*R*_ and *S*_*STE*_. However, as for the *S*_*STE*_, the absolute values of PCC are less than 0.5, with makes the results inconclusive. In order to extract more reliable information, we carried out further investigation by using supervised machine learning.

### Machine learning modeling

We employed four types of supervised machine learning models: Decision Tree Regression (DTR), Elastic Net (EN), Quadratic Polynomial LASSO (QP-LASSO), and Neural Network (NN)^31^. The DTR and EN models are constrained to only simple dependences, but they are straightforward to apply and can be used to extract dominant descriptors. In contrast, the NN model is very flexible and can reproduce a highly non-linear dependency, but it is much more difficult to interpret and prone to over-fitting. The QP-LASSO is in between NN and EN in terms of complexity and interpretability. In order to reduce the risk of over-fitting the available experimental data, we fix the number of descriptors to four, namely Δ*a, n*_*R*_*, S*_*R*_, and *L*_*R*_. In the Methods section, we discuss how these parameters were chosen out of a large set (#) of possible descriptors.

**Figure 2:**
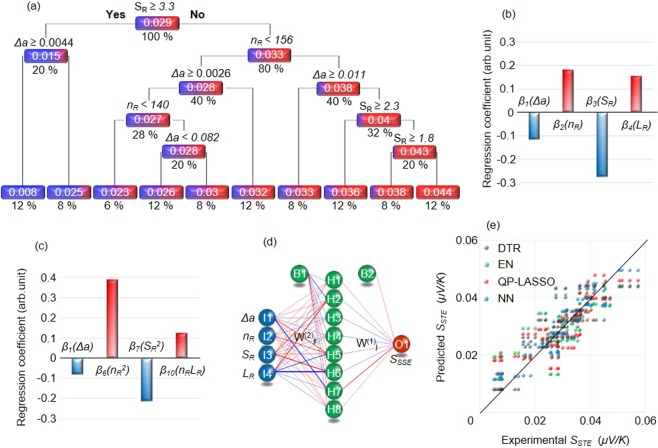
Informatics approach. **(a)** Visualization of the decision tree regression (DTR). Δ*a* and *S*_*R*_ are negatively correlated with *S*_*STE*_, while the *n*_*R*_ have positive correlation with *S*_*STE*_. (**b**) Regression coefficients for the elastic net (EN) model. The value of constant term *β*_0_ is 0.3310662. Δ*a* and *S*_*R*_ are negatively correlated with *S*_*STE*_, while the *n*_*R*_ and *L*_*R*_ have positive correlation with *S*_*STE*_, (**c**) Regression coefficient in the quadratic polynomial LASSO (QP-LASSO). The value of *β*_0_ is 0.3039554. Δ*a* and ${S}_{R}^{2}$ are negatively correlated with *S*_*STE*_, while the ${n}_{{R}}^{2}$ and *n*_*R*_*L*_*R*_ have positive correlation with *S*_*STE*_, (**d**) Visual representation of the neural network (NN) model. The line width represents the connection strength between units. Red/blue color demonstrate positive/negative correlation. (**e**) Predicted vs. measured values of *S*_*STE*_ for the DTR, EN, QP-LASSO and NN models. The cross validation error of the DTR, EN, QP-LASSO and NN are 8.56 × 10^−2^, 8.80 × 10^−2^, 8.55 × 10^−2^ and 5.52 × 10^−2^, respectively.

We start by constructing a DTR model, which predicts a target variable – in this case *S*_*STE*_ – by learning a series of simple decision rules based on the descriptors (Δ*a*, *n*_*R*_, *S*_*R*_, and *L*_*R*_). Figure 2a shows a visualization of the DTR model. Simple decision rules based on inequalities sort the data points quite accurately. Note that in the figure numbers in white and percentages denote the average *S*_*STE*_ values and the proportion of data points for each group, respectively. By observing the DTR model we can directly infer the relation between *S*_*STE*_ and the descriptors. Smaller *S*_*R*_, smaller Δ*a* and larger *n*_*R*_ lead to large *S*_*STE*_ in the DTR model. However, we were not able to obtain a relationship between *S*_*STE*_ and *L*_*R*_ through this model.

As a next step, we constructed a generalized linear model, EN, which is a combination of Ridge and LASSO regressions. This method assumes linear relationship between *S*_*STE*_ and the descriptors:

1 $${S}_{STE}({\rm{\Delta }}a,\,{n}_{R},\,{S}_{R},\,{L}_{R})={\beta }_{0}+{\beta }_{1}{\rm{\Delta }}a+{\beta }_{2}{n}_{R}+{\beta }_{3}{S}_{R}+{\beta }_{4}{L}_{R}$$

Although linearity can be an unnecessarily strong assumption, it helps minimize over-fitting. Figure 2b shows the values of *β*_1_*, β*_2_*, β*_3_ and *β*_4_ obtained as a result of the regression fit. We can directly interpret the relationship between descriptors and *S*_*STE*_. The *n*_*R*_ and *L*_*R*_ have positive correlation with respect to *S*_*STE*,_ while the *Δa* and *S*_*R*_ have negative correlation with *S*_*STE*_. However, DTR and especially EN models are very constrained and preclude proper modeling of more complicated dependencies. Therefore, to verify the patterns found by the EN, non-linear regression analysis using quadratic polynomial LASSO (QP-LASSO) model is applied next. We expand the linear model in equation 1 into a quadratic one:

2 $$\begin{array}{rcl}{S}_{STE}({\rm{\Delta }}a,\,{n}_{R},\,{S}_{R},\,{L}_{R}) & = & {\beta }_{0}+{\beta }_{1}{\rm{\Delta }}a+{\beta }_{2}{n}_{R}+{\beta }_{3}{S}_{R}+{\beta }_{4}{L}_{R}\\  &  & +{\beta }_{5}{\rm{\Delta }}{a}^{2}+{\beta }_{6}{n}_{R}^{2}+{\beta }_{7}{S}_{n}^{2}+{\beta }_{8}{L}_{n}^{2}\\  &  & +{\beta }_{9}{n}_{R}{S}_{R}+{\beta }_{10}{n}_{R}{L}_{R}+{\beta }_{11}{S}_{R}{L}_{R}\end{array}$$

(Note that interaction terms between the extrinsic (*Δa*) and the intrinsic (*n*_*R*_*, S*_*R*_*, L*_*R*_) factors with respect to the R element were deliberately not included.) QP-LASSO performs descriptor selection by adding *L*_1_ regularization term. This term tends to suppress the coefficients (*β*_0_, …., *β*_11_), and as a result only the ones in front of the most significant descriptors remain finite. Here, the QP-LASSO automatically selected four important descriptors: Δ*a, n*_*R*_^2^, *S*_*R*_^2^ *and n*_*R*_*L*_*R*_. The values of these coefficients - *β*_1_, *β*_6_, *β*_7_ and *β*_10_ - are shown in Fig. 2c. The *n*_*R*_ and *n*_*R*_*L*_*R*_ terms are positively correlated with *S*_*STE*_, while the coefficients in front of *Δa* and *S*_*R*_^2^ are negative. This agrees with the conclusions of the EN models.

The fourth algorithm we use is the NN - by far the most flexible of the four machine learning models we have employed. The flexibility comes at the price of significant risk of over-fitting, as well as difficulties interpreting the results. Figure 2d shows a visualization of the NN modeling result. By investigating the complex connections between the nodes (balls) carefully, we can find that the *S*_*STE*_ increases with increasing of *n*_*R*_ and *L*_*R*_, while the *S*_*STE*_ increases with decreasing of Δ*a* and *S*_*R*_. The details are shown in the Supplementary Information 2.

Figure 2e shows the accuracy of DTR, EN, QP-LASSO, and NN models. The horizontal and vertical axes are the values of *S*_*STE*_ measured in the experiments and those predicted by the machine learning models, respectively. We see that the NN model has better accuracy than the DTR, EN and QP-LASSO models, due to its much higher complexity. On the other hand, although the accuracy of the DTR, EN and the QP-LASSO models is not as high, interpreting their implications is much more straightforward. Despite these differences, all four machine learning algorithms converge on a similar picture in which *S*_*STE*_ is positively correlated with *n*_*R*_ and *L*_*R*,_ while negatively correlated with *Δa* and *S*_*R*_.

The correlations between *S*_*STE*_ and Δ*a*, *n*_*R*_, and *S*_*R*_ can be explained based on the conventional understanding of the STE phenomena. However, the positive correlation between *L*_*R*_ and *S*_*STE*_ uncovered by the machine learning models appears to be beyond our current knowledge of STE. (The details of the physical interpretation underlying these relations are discussed in Supplementary Information 3). The surprising connection between *S*_*STE*_ and *L*_*R*_, discovered by the machine learning models here, can lead to a more comprehensive understanding of the mechanism of STE.

### Development of a superior STE material

We now demonstrate that the unanticipated results of the machine learning modelling can indeed help us develop improved STE materials. We use the positive correlation between *L*_*R*_ and *S*_*STE*_ to search for other STE materials relying on anomalous Nernst effect (ANE). The SSE and the ANE are distinct but similar spin-driven thermoelectric (STE) phenomena, both originating in the spin-orbit interaction. Thus, it is reasonable to conjecture that tuning the orbital angular momentum *L*_*R*_ can enhance not only the SSE but also the ANE. In conventional STE materials utilizing ANE, FePt alloy has exhibited the largest thermopower so far^32^. Therefore, we expect that adding rare-earth elements R with *L*_*R*_ into Fe-Pt alloy will increase the thermopower. As an initial example, we investigate the thermopower of Fe-Pt-Sm ternary alloy, where we have selected Sm as one of the R elements with large *L*_*R*_.

**Figure 3:**
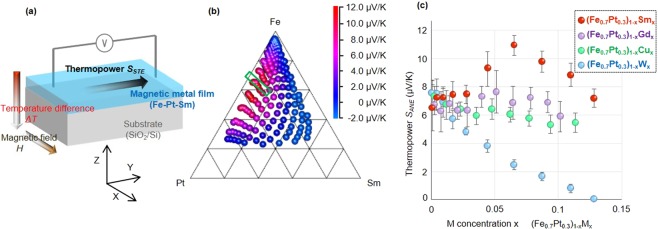
Development of better STE material using ANE. **(a**) Schematic of the spin-driven thermoelectric (STE) devise using anomalous Nernst effect (ANE) consisting of a Fe-Pt-Sm layer and SiO_2_/Si substrate. (**b**) Thermopower *S*_*ANE*_ of Fe-Pt-Sm on SiO_2_/Si as a function of composition data. (**c**) Thermopower *S*_*STE*_ of (Fe_0.7_Pt_0.3_)_1−x_M_x_ on SiO_2_/Si as a function of composition data. Error bars show standard deviations.

Figure 3a shows the general configuration of the STE device using ANE. When a temperature difference Δ*T* and a magnetic field *H* are applied along the z and the x direction, respectively, one can detect the thermopower *S*_*STE*_ along the y direction, just like the case of SSE shown in Fig. 1a. In order to optimize the composition within the ternary alloy, various Fe-Pt-Sm alloy with different composition were investigated. The details of this device are provided in the Supplementary Information 4.

Figure 3b shows the measured *S*_*STE*_ values for the Fe-Pt-Sm as a function of composition. The largest *S*_*STE*_ was detected near composition Fe_0.7_Pt_0.3_Sm_0.05_. To further investigate the region outlined by the green rectangle in greater detail and to confirm the contribution of *L*_*R*_ (Sm), we investigated the thermopower of (Fe_0.7_Pt_0.3_)_1−x_M_x_ alloy with small amount of different M atoms (M = Sm, Gd, Cu and W). Of these elements only Sm has finite orbital angular moment_,_ while the other have *L*_*R*_ = 0. Figure 3c shows the *S*_*STE*_ of the (Fe_0.7_Pt_0.3_)_1−x_M_x_. It is clear that a small amount of Sm is necessary to maximize *S*_*STE*_. With Gd, Cu and W, there is no *S*_*STE*_ enhancement, thus confirming the crucial role of large *L*_*R*_.

## Discussion

To benchmark the *S*_*STE*_ of the Fe-Pt-Sm material, we compare it with those of other STE materials with ANE^32^. According to Ikhlas *et al., S*_*STE*_ of most ferromagnetic materials are on the order of 0.1 μV/K. In comparison, the largest *S*_*STE*_ obtained here (11.12 μV/K of Fe_0.665_Pt_0.27_Sm_0.065_ in Fig. 3c) is at least one order of magnitude larger than those of other known ANE materials. It is interesting to note that while some of the highest *S*_*STE*_ values of previously known materials consist of Fe and Pt at 50/50 concentration (Pt/Fe multilayers and L1_0_ FePt with *S*_*STE*_ ≤ 1 μV/K), our investigation shows that the optimum Fe-Pt occurs at around Fe_0.7_Pt_0.3_ (with *S*_*ANE*_ of ≈7 μV/K) which is then further enhanced by Sm substitution as discussed above.

An STE device has two possible large-scale applications – thermoelectric generation^4^ and heat flow sensing^5^. Thermoelectric generators enable the reuse of ubiquitous and wasted heat energy, and are becoming indispensable components in energy harvesting systems. Heat flow sensors, on another hand, can be used in smart thermal management systems for mapping the thermal energy flow. In order to make STE devices practical for either of these applications, significant improvement of their thermopower is necessary. The identification of a novel material with dramatically enhanced thermopower reported here demonstrates that such improvements are possible, and STE technologies present a viable way towards a more energy-efficient future.

In summary, we have demonstrated the utility of machine learning both in exploring the fundamental physics of the STE phenomena and in optimizing the materials harnessing these effects. Using a data-driven approach has allowed us to construct unbiased statistical models for STE, which led us to a materials design rule, not rooted in the conventional theory of STE. Combining it with experimentation we have discovered an STE material with *S*_*STE*_ an order of magnitude larger than that of any previously known ANE material. Thus, machine learning was the key to an important step in turning STE into a practical and affordable technology.

## Methods

### Fabrication of STE devices using SSE (Pt/R:YIG/GGG or SGGG)

The fabrication method for the STE devices using SSE followed two steps. First, R:YIG layer was formed on the substrate (GGG or SGGG, 500 μm thickness) by means of the metal-organic-decomposition (MOD) method^33^. The MOD solution includes R, Y and Fe carboxylate, dissolved in organic solvents. Its chemical composition is R:Y:Fe = 1:2:5. The MOD solution was spin-coated on substrate at 1000 r.p.m. for 30 second, and then dried at 150 °C for 5 minutes. After pre-annealed at 450 °C for 5 minutes, it was annealed at 700 °C for 14 hours in air, to form a crystallized R:YIG layer. Its thickness was estimated to be 60 nm from the interference thickness meter. After completion of the R:YIG layer, a 10-nm-thickness Pt layer was deposited on the R:YIG layer by sputtering. For the measurement, the devices was cut into small chips, the length and width of which were 8.0 mm and 2.0 mm respectively.

### Fabrication of STE device using ANE (Fe-Pt-Sm/SiO_2_/Si)

The STE devices using ANE were fabricated as follows. The Fe-Pt-Sm film with composition gradient was deposited on 3 inch SiO_2_/Si wafer by combinatorial sputtering at room temperature. The thickness of Fe-Pt-Sm, SiO_2_ and Si layer are150 nm, 0.5 μm and 381 μm, respectively. For the measurement, it was cut into small chips, with length and width identical to those cut from the Pt/R:YIG film.

### Measurement for the STE thermopower *S*_*STE*_

A temperature difference ΔT directed along the z direction as shown in Figs 1a and 3a was applied between the top and the bottom of the devices, by sandwiching them between copper heat bath at 300 K and 300 + ΔT K. The magnetic field *H* was applied along the x direction. Under these conditions, the STE thermopower can be detected along the y direction. The distance between voltage-detection terminals were set to 6 mm.

### Selecting descriptors and hyper-parameters for the machine learning models

One major issue in developing machine learning models is avoiding overfitting. As a general rule, when the amount of available data is small, the number of descriptors should be constrained. For example, Seko *et al*.^34^ employed machine learning to predict melting temperature, with the number of data points (compounds) and descriptors (predictors) 248 and 10, respectively. In our case there are only 112 data points (see Fig. 2e) and therefore it is advisable to use an even smaller number of descriptors. We have considered a number of descriptors, covering different properties of the rare-earth elements. These include atomic weight, spin and orbital angular momenta, number of unfilled orbitals, melting temperatures, magnetic moments, volumes and space groups (the last four are calculated for the elemental ground state). Magpie software was used to generate some of these^35^. For data pre-processing, we calculated PCC in order to detect multicollinearity. Almost all of the descriptors calculated by the Magpie software have high PCC value with respect to either *Δa, n*_*R*_*, S*_*R*_ or *L*_*R*_. They are easy to interpret and connect to STE phenomenology, and at the same time models build with only these have accuracy comparable to that of models utilizing the full list of descriptors. In the future, we hope to increase the size of the experimental data and thus be able to include more descriptors in the model.

The hyper-parameters of the models are decided with the help of Leave-Out-One Cross-Validation (LOOCV) – a widely used model validation technique. In this scheme one data point is retained as validation data for testing the model, while the rest of the dataset is used as training data. The hyper parameters of the model are determined by minimizing the error indicator such as root mean square error (RMSE) or mean square error (MSE) on the test point. In this paper, we used RMSE as the error indicator. The LOOCV was carried out by “caret” package in R programming language.

### Decision tree regression (DTR)

The Decision Tree Regression is a non-parametric machine learning model based on a series of simple decision rules, which combine flexibility with interpretability. The only model hyperparameter – complex parameter (cp) – was set to 3.90625 × 10^−3^ by LOOCV, with cross validation error of 8.560104 × 10^−2^. The DTR was carried out by “rpart” package in R programming language.

### Elastic Net (EN)

The Elastic Net is a generalized linear model, combination of Ridge and Lasso regressions. The mixing ratio of the Ridge and the Lasso (Ridge: Lasso) was set to 1: 0 based on the LOOCV. Therefore, in our case the EN model was equivalent to a Ridge regression. The LOOCV also decided the magnitude of generalization (λ: 3.90625 × 10^−3^), and the cross validation RMSE was 8.798218 × 10^−2^. The EN was carried out by “glmnet” package in R programming language.

### Quadratic polynomial least absolute shrinkage and selection operator (QP-LASSO)

The LASSO is a regression analysis method that performs both variable selection and regularization. The QP-LASSO selects among quadratic, linear and constant terms. In this case QP-LASSO selected four valuables, including Δ*a, n*_*R*_^2^*, S*_*R*_^2^ *and n*_*R*_*L*_*R*_, from equation (2). The LOOCV-determined magnitude of generalization is (λ: 7.55559 × 10^−3^), and the cross validation RMSE is 8.547411 × 10^−2^. The QP-LASSO was carried out by “glmnet” package in R programming language.

### Neural Network (NN)

The NN method models the data by means of a statistical learning algorithm mimicking the brain. Here we have utilized simple 3-layer perceptron NN, with the number of input units, hidden units and output units being 4, 8 and 1, respectively. The hidden units and the output unit simulate the activation of a neuron by applying the hyperbolic tangent and the sigmoid functions, respectively. Mathematically, the NN models the non-linear function *S*_*STE*_ (Δ*a, n*_*R*_*, S*_*R*_*, L*_*R*_) by performing the following calculation.

$${S}_{STE}({\rm{\Delta }}a,\,{n}_{R},\,{S}_{R},\,{L}_{R})={S}_{STE}(x,\,w)=\sigma (\sum _{j=0}^{8}\,{w}_{j}^{(2)}h(\sum _{i=0}^{4}\,{w}_{ij}^{(1)}{x}_{i}))$$

The *x*_1_, *x*_2_, *x*_3_, *x*_4_, *h* and *σ* are *Δa*, *n*_*R*_, *S*_*R*_, *L*_*R*_, hyperbolic tangent function and sigmoid function, respectively. The weights and the bias parameters ${W}_{j}^{2}$ and ${W}_{ji}^{2}$ are determined by minimizing the cost function with the backpropagation algorithm. A decay value was set to 1.220703 × 10^−4^. The hyper parameters, such as the number of hidden units and the value of the decay were decided by LOOCV, and cross validation RMSE of 5.516461 × 10^−2^ was achieved. For NN analysis, we used “nnet” package in R programming language.

## Supplementary information

Machine-learning guided discovery of a new thermoelectric material

## Data Availability

The data and the code that support the results within this paper and other findings of this study are available from the corresponding author upon reasonable request.

## References

[1] Rowe, D. M. *CRC Handbook of Thermoelectrics: Macro to Nano* (CRC Press, 2005).

[2] Goldsmid, H. J. *Introduction to Thermoelectricity* (Springer, 2010).

[3] Bell LE (2008). Cooling, heating, generating power, and recovering waste heat with thermoelectric systems. Science.

[4] Kirihara A (2012). Spin-current-driven thermoelectric coating. Nature mater..

[5] Kirihara A (2016). Flexible heat-flow sensing sheets based on the longitudinal spin Seebeck effect using one-dimensional spin-current conducting films. Sci. Rep..

[6] Bauer GEW, Saitoh E, van Wees BJ (2012). Spin caloritronics. Nature mater..

[7] Uchida K (2008). Observation of the spin-Seebeck effect. Nature.

[8] Uchida K (2010). Spin Seebeck insulator. Nature Mater..

[9] Uchida K (2014). Longitudinal spin Seebeck effect: from fundamentals to applications. J. Phys.: Condens. Matter.

[10] Uchida K, Nonaka T, Ota T, Saitoh E (2010). Longitudinal spin-Seebeck effect in sintered polycrystalline (Mn, Zn)Fe_2_O4. Appl. Phys. Lett..

[11] Huang SY, Wang WG, Lee SF, Kwo J, Chien CL (2011). Intrinsic Spin-Dependent Thermal Transport. Phys. Rev. Lett..

[12] Sakuraba Y (2016). Potential of thermoelectric power generation using anomalous Nernst effect in magnetic materials. Scr. Mater..

[13] Taniguchi T (2016). Phenomenological spin transport theory driven by anomalous Nernst effect. J. Phys. Soc. Jpn..

[14] Azevedo A, Vilela Leao LH, Rodriguez-Suarez RL, Oliveira AB, Rezande SM (2005). Dc effect in ferromagnetic resonance: Evidence of the spin-pumping effect. J. Appl. Phys..

[15] Saitoh E, Ueda M, Miyajima H, Tatara G (2006). Conversion of spin current into charge current at room temperature: Inverse spin-Hall effect. Appl. Phys. Lett..

[16] Costache MV, Sladkov M, Watts SM, van der Wal, van Wees BJ (2006). Electrical Detection of Spin Pumping due to the Precessing Magnetization of a Single Ferromagnet. Phys. Rev. Lett..

[17] Valenzuela MV, Tinkham M (2006). Direct electronic measurement of the spin Hall effect. Nature.

[18] Tikhonov KS, Sinova J, Finkel’stein AM (2013). Spectral non-uniform temperature and non-local heat transfer in the spin Seebeck effect. Nature Commun..

[19] Adachi H (2010). Gigantic enhancement of spin seebeck effect by phonon drag. Appl. Phys. Lett..

[20] Butler KT, Davies DW, Cartwright H, Isayev O, Walsh A (2018). Machine learning for molecular and materials science. Nature.

[21] Mueller, T., Kusne, A. G. & Ramprasad, R. Machine learning in material science: recent progress and emerging applications. *Reviews in Computational Chemistry* (2016)

[22] Kusne AG (2014). On-the-fly machine-learning for high-throughput experiments: search for rare-earth-free permanent magnets. Sci. Rep..

[23] Balachandran PV, Young J, Lookman T, Rondinelli JM (2017). Learning from data to design functional materials without inversion symmetry. Nat. Comm..

[24] Stanev V (2018). Machine learning modeling of superconducting critical temperature. npj comput. Mater..

[25] Nikolaev P (2016). Autonomy in materials research: a case study in carbon nanotube growth. npj Comput. Mater..

[26] Koinuma H, Takeuchi I (2004). Combinatorial solid-state chemistry of inorganic materials. Nat. Mater..

[27] Takeuchi I (2006). Combinatorial experiment and materials informatics. MRS Bull..

[28] Takeuchi I (2003). Identification of novel compositions of ferromagnetic shape-memory alloys using composition spreads. Nat. Mater..

[29] Takeuchi I (2003). Monolithic multichannel ultraviolet detector arrays and continuous phase evolution in Mg_x_Zn_1−x_O composition spreads. J. Appl. Phys..

[30] Iwasaki Y, Kusne AG, Takeuchi I (2017). Comparison of dissimilarity measures for cluster analysis of X-ray diffraction data from combinatorial libraries. npj Comput. Mater..

[31] Bishop, C. M. Pattern Recognition and Machine Leaning (Springer, 2006).

[32] Ikhlas M (2017). Large anomalous Nernst effect at room temperature in a chiral antiferromagnet. Nat. Phys..

[33] Ishibashi T (2005). Characterization of epitaxial (Y,Bi)_3_(Fe,Ga)_5_O_12_ thin films grown by metal-organic decomposition method. J. Appl. Phys..

[34] Seko A, Maekawa T, Tsuda K, Tanaka I (2014). Machine learning with systematic density-function theory calculations: Application to melting temperature of single- and binary- component solids. Phys. Rev. B.

[35] Ward L (2016). A general-purpose machine learning framework for predicting properties of inorganic materials, npj Computational. Materials.

