# Supplementary material for: Machine-learning guided discovery of a new thermoelectric material
Source: Sci Rep. 2019 Feb 26;9:2751. doi: 10.1038/s41598-019-39278-z (PMC6391459; doi:10.1038/s41598-019-39278-z)
Supplement: Supplementary file 1 — Machine-learning guided discovery of a new thermoelectric material [file 41598_2019_39278_MOESM1_ESM.docx]

**Supplemental Information**

**Machine-learning guided discovery of a new thermoelectric material**

Yuma Iwasaki^1,2^*, Ichiro Takeuchi^3,4^, Valentin Stanev^3,4^, Aaron Gilad Kusne^3,5^, Masahiko Ishida^1^, Akihiro Kirihara^1^, Kazuki Ihara^1^, Ryohto Sawada^1^, Koichi Terashima^1^, Hiroko Someya^1^,Ken-ichi Uchida^2,6,7,8,9^, Eiji Saitoh^8,9,10,11^ , Shinichi Yorozu^1^

*^1^Central Research Laboratories, NEC Corporation, Tsukuba 305-8501, Japan*

*^2^PRESTO, JST, Saitama 322-0012, Japan*

*^3^Department of Materials Science and Engineering, University of Maryland, College Park, MD 20742, USA*

*^4^Center for Nanophysics and Advanced Materials, University of Maryland, College Park, MD 20742, USA*

*^5^National Institute of Standards and Technology, Gaithersburg, MD 20899, USA*

^6^Research Center for Magnetic and Spintronic Materials (CMSM), National Institute for Materials Science (NIMS), Tsukuba, 305-0047, Japan

^7^Research and Services Division of Materials Data and Integrated System (MaDIS), National Institute for Materials Science (NIMS), Tsukuba, 305-0047, Japan

*^8^Institute for Materials Research, Tohoku University, Sendai 908-8577, Japan*

*^9^Center for Spintronics Research Network, Tohoku University, Sendai, 980-8577, Japan*

*^10^Advanced Institute for Materials Research, Tohoku University, Sendai 908-8577, Japan*

*^11^Advanced Science Research Center, Japan Atomic Energy Agency, Tokai, 319-1195, Japan*

Email: [y-iwasaki@ih.jp.nec.com](mailto:y-iwasaki@ih.jp.nec.com)

**1. STE device using SSE**

Schematic of the spin-driven thermoelectric (STE) device using the spin-Seebeck effect (SSE) ^6-10^ is shown in Supplemental Figure 1a. It is composed of a paramagnetic conductive layer, a magnetic layer, and a single crystal substrate. We adopted a bilayer consisting of platinum (Pt) and rare-earth-substituted yttrium iron garnet (R_1_Y_2_Fe_5_O_12_, referred to as R:YIG), where R stands for a rare-earth element. The YIG and Pt are believed to be a good combination for STE conversion due to their long magnon-diffusion length (*D_L_*) and large spin-orbit interaction ^4^. The typical thickness of the R:YIG layer is 60 nm. Since strain in the films is known to influence spin transport in materials and because the lattice constant of R:YIG depends on the choice of R, two substrates were used: a (111)-oriented Gadolinium Gallium Garnet (Gd_3_Ga_5_O_12_, referred to as GGG, with the lattice constant *a* = 12.385 å) and a (111)-oriented Substituted Gadolinium Gallium Garnet (Gd_2.675_Ca_0.325_Ga_4.025_Mg_0.325_Zr_0.65_O_12_, referred to as SGGG, with the lattice constant *a* = 12.464 å). Thus, different R:YIG layers have different degrees of lattice match to the two substrates. Supplemental Figure 1b shows the cross-sectional image of a typical device observed by transmission electron microscopy. The epitaxial YIG layer with a coherent interface was formed on the SGGG substrate. When a temperature difference *ΔT* and a magnetic field *H* are applied along the z and x direction, respectively, a spin current is generated along the z direction by the SSE in the R:YIG layer (Supplemental Figure. 1a). The spin current is then injected into the Pt layer and converted into an electric current by the Inverse spin Hall effect (ISHE) ^14-17^. One then detects the thermoelectric voltage V_SSE_ (SSE voltage, *V_SSE_*) along the y direction. Supplemental Figure 1c shows typical *V_SSE_* behaviors as a function of *H* for different *ΔT*. Note that the sign of *V_SSE_* changes when the sign of *H* is inverted - a clear indication that the thermoelectric voltage arises from the SSE and the ISHE. The thermopower *S_STE_* is calculated as $S_{STE}=\left( {V_{SSE}}/{\Delta T} \right)\left( {L_{z}}/{L_{y}} \right)$ ^36^.


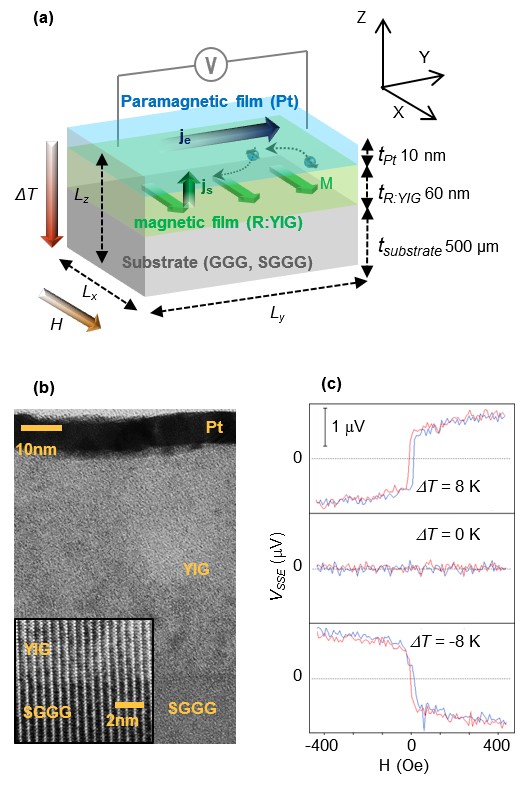


**Supplemental Figure 1 | A STE device using SSE.** **a**, Schematic of the STE device consisting of a Pt layer, a rare-earth substituted yttrium iron garnet (R_1_Y_2_Fe_5_O_12_, referred to as R:YIG) layer and a (111)-oriented Gadolinium Gallium Garnet (Gd_3_Ga_5_O_12_, referred to as GGG) substrate or a (111)-oriented Substituted Gadolinium Gallium Garnet (Gd_2.675_Ca_0.325_Ga_4.025_Mg_0.325_Zr_0.65_O_12_, referred to as SGGG) substrate. Spin current *j_s_* generated from heat current in the magnetic layer by the spin-Seebeck effect is converted into electrical current *j_e_* by the inverse spin Hall effect in the Pt layer. **b,** Cross-section transmission electron microscopy image of a STE multilayer structure (Pt/YIG/SGGG). The inset shows that the magnetic layer has grown epitaxially on the substrate. **c,** The spin-Seebeck voltage *V_SSE_* of a spin-Seebeck device (Pt/YIG/SGGG) as a function of *H*. The sign of *V_SSE_* follows the sign of *H*, indicating that the thermo-electromotive force arises from the spin-Seebeck effect and the inverse spin-Hall effect.

**2. Interpretation of neural network (NN) model**

The fourth algorithm we use is the NN - by far the most flexible of the four machine learning models we have employed. This comes at the price of significant risk of over-fitting, as well as difficulties interpreting the results. Figure 2d shows a visualization of the NN modeling result. It has input units (I, blue balls), hidden units (H, green balls), bias units (B, green balls) and an output unit (O, red balls). The strength of the dependence between units is represented as line width, with larger width indicating stronger connection. The red and blue lines denote positive and negative correlation between units, respectively. The relationship between input and output units is expressed as the product of the input-hidden correlation and hidden-output correlation. For example, l1-H4-O1 correlation is positive, because both l1-H4 (blue) and H4-O1 (blue) correlations are negative.

The visualization in Figure 2d provides a graphical summary of the relationship between the descriptors (the input units) and *S_STE_* (the output unit). For *Δa* (I1), the strongest path from I1 to O1 is l1-H7-O1 path, where l1-H7 connection is negative while H7-O1 connection is positive. This implies a negative correlation between *Δa* and *S_STE_*. In case of *n_R_* (l2), the strongest path is I2-H5-O1 and both I2-H5 and H5-O1 connections are positive. Therefore, the relation between *n_R_* (l2) and *S_STE_* is positive. For *S_R_* (l3), the strongest path from l3 to O1 is I3-H6-O1. This indicates overall negative correlation between *S_R_* (l3) and *S_STE_* because I3-H6 connection is positive while H6-O1 is negative. Similarly, the relation between *L_R_* (l4) and *S_STE_* is positive because of the strongest I4-H6-O2 path of which both I4-H6 and H6-O1 connections are negative.

**3. Physical and theoretical interpretation of correlations obtain from machine learning**

The positive correlation between *n_R_* and *S_STE_* can be understood by considering the thermal conductivity *κ* of the R:YIG film. *S_STE_* is proportional to the temperature difference *ΔT* over the R:YIG layer ^37^, which in our setup is inversely proportional to *κ. κ* is known to decrease with increasing *n_R_*_,_ due to the enhanced phonon scattering ^38^.The negative correlation between *S_R_* and *S_STE_* can be readily explained by considering *M_s_*. The saturation magnetization decreases with increasing *S_R_* because the spin magnetic moment *S_R_* in R:YIG is arranged to cancel *M_s_* ^39^. It is also known that the relation between *M_s_* and *S_STE_* is positive: larger *M_s_* leads to larger spin currents ^40^. Therefore, increasing *S_R_* lead to a drop in *S_STE_*. The negative correlation between *Δa* and *S_STE_* can be explained with the crystalline quality of R:YIG films. In general, larger lattice mismatch leads to worse crystallinity (through strain and defects), which in turn decreases the magnon (spin-current) diffusion length *D_L_* in the R:YIG film. In previous work it was found that *S_STE_* decreases with decreasing *D_L_* ^41^. It is thus straightforward to see how a larger *Δa* (which results in local crystalline defects such as dislocations in the R:YIG layer) leads to a reduction in *S_STE_.*

Thus, the machine-learning derived correlations between *S_STE_* and *Δa*, *n_R_,* and *S_R_* can be explained based on the conventional understanding of the SSE physics. However, the positive correlation between *L_R_* and *S_STE_* uncovered by the machine learning models appears to be beyond our current knowledge of SSE. The fact that increasing *L_R_* leads to increasing heat-to-spin current conversion efficiency cannot be easy reconciled with the magnon-driven theory of SSE ^42^, which has gained broad acceptance recently. In fact, within this theory, the opposite trend is expected; larger *L_R_* was believed to result in increase in the spin-phonon interaction, and accordingly reduction in magnon diffusion length ^43^ and thus smaller *S_STE_.* The positive correlation between *L_R_* and *S_STE_* is more consistent with the phonon-driven SSE theory ^18,19^, where the phonon-mediated SSE may be enhanced by the large spin-phonon interaction due to *L_R_*. In other words, increasing *L_R_* might increase the phonon contribution to the spin current generation. The surprising connection between *S_STE_* and *L_R_*, discovered by the machine learning models here, can perhaps lead to a more comprehensive description of the mechanism of SSE in the future.

**4. STE device using ANE and combinatorial experiment**

Supplemental Figure 2a shows a schematic of the spin-driven thermoelectric (STE) device using anomalous Nernst effect (ANE). It is composed of a magnetic metal layer (Fe-Pt-Sm) and a substrate (SiO_2_/Si). We apply the temperature difference *ΔT* and a magnetic field *H* are applied along the z and x direction, respectively, and measure the thermopower *S_STE_* along the y direction in the same manner as the above-described STE device using SSE. In order to optimize the composition within the ternary, we have carried out a combinatorial experiment ^26-30^ using a thin-film composition spread on SiO_2_/Si , mapping a large fraction of the Fe-Pt-Sm ternary on one library wafer (Supplemental Figure 2b, 2c). The thin film sample was then separated (Supplemental Figure 2a) in order to measure the *S_STE_*.


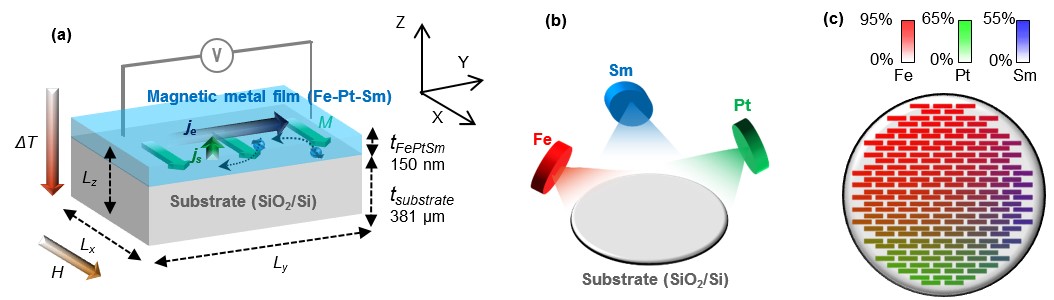


**Supplemental Figure 2 | STE device using ANE and combinatorial experiment.** **a.** Schematic of the spin-driven thermoelectric (STE) device using the anomalous Nernst effect (ANE) consisting of a Fe-Pt-Sm layer and SiO_2_/Si substrate. **b.** Schematic illustration of combinatorial sputtering method. **c.** Composition spread mapping of Fe-Pt-Sm film fabricated by combinatorial sputtering.

**Reference**

36. Uchida, K. et al. Thermoelectric Generation Based on Spin Seebeck Effects. Proc. IEEE **104,** 1946 (2016)

37. Kikkawa, T. et al. Separation of longitudinal spin Seebeck effect from anomalous Nernst effect: Determination of origin of transverse thermoelectric voltage in metal/insulator junctions. Phys. Rev. B **88,** 214403 (2013)

38. Gaume, R., Viana, B., Vivien, D., Roger, J. P. & Fournier. D. A simple model for the prediction of thermal conductivity in pure and doped insulating crystals. Appl. Phys. Lett. **83,** 1355 (2003)

39. Sekijima, T., Kishimoto, H., Fujii, T., Wakino, K. & Okada, M. Magnetic, optical and microwave properties of rare-earth-substituted fibrous yttrium iron garnet single crystals grown by floating zone method. Jpn. J. Appl. Phys. **38,** 5874-5878 (1999).

40. Uchida, K., Nonaka, T., Kikkawa, T., Kajiwara, T. & Saitoh, E. Longitudinal spin Seebeck effect in various garnet ferrite. Phys. Rev. B **87,** 104412 (2013)

41. Rezende, S. M. et al. Magnon spin-current theory for the longitudinal spin-Seebeck effect. Phys. Rev. B **89,** 014416 (2014)

42. Xiao, J., Bauer, G. E. W., Uchida, K., Saitoh, E. & Maekawa, S. Theory of magnon-driven spin Seebeck effect. Phys. Rev. B **81,** 214418 (2010)
